# Estimating the out‐of‐pocket health expenditure in patients under 5 years with severe malnutrition in Afghanistan in 2023: Findings from a cross‐sectional study

**DOI:** 10.1002/hsr2.2256

**Published:** 2024-07-18

**Authors:** Amirmohammad Tajik, Vahid Ghavami, Shakib Popal, Hamidreza Shabanikiya, Mehdi Varmaghani

**Affiliations:** ^1^ School of Pharmacy Mashhad University of Medical Sciences Mashhad Iran; ^2^ Health Policy Research Center, Institute of Health Shiraz University of Medical Sciences Shiraz Iran; ^3^ Department of Biostatistics, School of Health Mashhad University of Medical Sciences Mashhad Iran; ^4^ General Directorate of Human Resources Ministry of Public Health Herat Afghanistan; ^5^ Social Determinants of Health Research Center Mashhad University of Medical Sciences Mashhad Iran; ^6^ Department of Health Economics and Management Sciences, School of Health Mashhad University of Medical Sciences Mashhad Iran

**Keywords:** Afghanistan, child, healthcare rationing, health equity, health expenditures, malnutrition

## Abstract

**Introduction:**

This study, of significant importance to healthcare professionals, policymakers, researchers, and organizations involved in child healthcare and malnutrition in Afghanistan, aimed to estimate the out‐of‐pocket expenditure (OOPE) in patients under 5 years old with severe malnutrition in a children's hospital in Herat Province, Afghanistan.

**Method:**

This study employed a meticulously designed cross‐sectional descriptive‐analytical approach with practical results. The research population consisted of families with malnourished children under 5 who were referred to Herat Children's Hospital. Data was collected using a comprehensive standard World Health Organization questionnaire to gather demographic information from children in Herat. A carefully selected convenience sampling method was used, with 300 referring patients participating in face‐to‐face interviews with the supervisors of these children. After obtaining personal consent and coordinating with health officials, interviews were conducted with the caregivers of children under 5 who suffered from severe malnutrition. The data was then analyzed using robust descriptive statistics, quantitative variables, mean and standard deviation, frequency, and relative frequency. Multiple regression analysis was used to determine the factors that most influenced direct payments from patients' pockets, ensuring the reliability and validity of the findings.

**Results:**

The results showed that OOPE in both households with seven and less than seven people and more than seven people was 68%. The findings indicated that among the residents of Herat referred to the studied hospital, these people spent 54% of the treatment costs directly out of pocket. In contrast, people in the rural areas of Herat pay 69% of the treatment costs to receive medical services straight out of pocket. The critical point is that 93% of the families have incurred catastrophic expenses to treat their children suffering from severe malnutrition. The research revealed that the patient's location and the education level of the head of the household were the most significant factors affecting out‐of‐pocket payments by patients.

**Conclusion:**

Increasing OOPE in rural Afghanistan poses a significant obstacle to equitable healthcare services and access to appropriate medicines. To support the goal of universal healthcare coverage, geographic imbalances, and broad health financing options must be addressed. Strengthening insurance coverage and more government assistance can significantly reduce these patients' out‐of‐pocket payments.

## 1 INTRODUCTION

Most people believe that in today's world, due to demographic changes and patterns of new diseases, health is one of the essential components of quality of life. For this reason, the role of health services is significant in maintaining this critical role.^1^ But due to economic crises, lack of resources for health systems, new medical technologies, and their high cost, much money is imposed on health systems and people; so that people have been forced not to seek and not use health services due to lack of financial ability, as a result of which people get sick and lose their jobs and finally face financial problems.^2^ According to the World Health Organization (WHO) report in 2008, about 25 million households, more than 100 million people, are trapped in poverty due to paying these costs.^3^ A study also showed that in nine Southeast Asian countries, at least 30% of health financing was through direct payment from pocket.^4^

Therefore, the WHO has placed particular emphasis on protecting households against the costs of health services. The third main goal of the countries' health system has been justice in financing families. In line with justice, the WHO has put forward the Fair Financial Contribution Index, which measures fairness in distributing the financial burden of medical expenses in society.^5^ A fair system is a system in which households participate in healthcare costs according to their economic and income capabilities and do not pay more than their ability to pay, which leads to catastrophic payments.^6^ Consumer payments are the costs people pay out‐of‐pocket expenditure (OOPE) directly when purchasing healthcare services. OOPE, which includes consultation fees, medicine costs, and transportation required during treatment, is a significant barrier to seeking healthcare.^7^, ^8^

In Afghanistan, according to the constitution, the government must provide health services and health centers free of charge to the public. Still, when people go to health centers, they experience problems. There are expenses, especially in the private sector, which are unknown to the general public, and their payment is demanding and catastrophic for some people.^9^ Severe malnutrition is one of the major problems of the health system in Afghanistan, which is one of the major contributors to the death of children under the age of five. The Afghanistan Multiple Indicator Cluster Survey (MICS) study conducted by the United Nations Children's Fund office in Afghanistan in 2023, as well as other studies, indicates that 41% of children under 5 years are struggling with stunting and 9.5% of children under 5 years of age in Afghanistan suffer from wasting, the extreme manifestation of severe malnutrition.^10^, ^11^ Undernutrition significantly increases the vulnerability of children to diseases and mortality. In lower‐ and middle‐income countries, approximately 45% of deaths in children under the age of five are associated with undernutrition.^9^ Severe malnutrition impacts more than 50 million children under the age of 5, contributing to 8.0% of global child deaths annually.^8^ Hence, prevention and rapid treatment are essential in this case.

A child's nutritional status is indicative of their overall health. Childhood undernutrition, leading to conditions such as wasting, fetal growth restriction, stunting, and micronutrient deficiencies, poses a significant burden on the health system and negatively impacts the economic and sociocultural fabric of society.^12^, ^13^, ^14^, ^15^ Poverty and malnutrition play an essential role in higher rates of morbidity and mortality, hinder cognitive development in children, and elevate the incidence of common childhood infections.^16^, ^17^, ^18^ Moreover, it diminishes economic productivity in adulthood. Severe malnutrition, a clinical term for undernutrition, is a significant concern in the health sector.^17^ It is defined as a weight‐for‐height *Z* score < − 3.

After an extensive review of literature from known databases, there is a scarcity of studies addressing the costs associated with Afghanistan's healthcare system and the out‐of‐pocket expenses by patients.^18^, ^19^ Considering no study was found evaluating out‐of‐pocket malnutrition patients in Afghanistan, this study marks the pioneering effort in Afghanistan to explore the impact of health costs imposed on families living in Afghanistan. So, the researchers focus on direct payment from the pocket of patients admitted to the malnutrition department in the children's hospital in Herat Province, Afghanistan. The children's hospital was chosen because it is the only specialized hospital in Herat Province. Patients admitted to Herat Children's Hospital because they belong to poor and low‐income groups and because most patients and groups who refer to this hospital come from different cities and neighboring provinces for treatment calculate direct out‐of‐pocket payments. They are essential for policy‐making in the field of therapy. Therefore, this study aims to calculate direct out‐of‐pocket costs in severely malnourished patients admitted to a children's hospital in Herat Province.

## 2 METHOD

The study was conducted cross‐sectionally and yielded practical results descriptively. The research population includes families with malnourished children under 5 referred to Herat Children's Hospital. For this purpose, the inclusion criteria were all patients hospitalized due to severe malnutrition with particular measures and willing to participate in the study (average arm size less than 115 mm or *Z* score less than minus 3 with edema +3 or other related disorders). Also, patients who did not want to continue cooperating in the study were considered exclusion criteria. According to the mentioned cases, a convenience sampling method was used with 300 referring patients utilizing face‐to‐face interviews with the supervisors of these children from hospitalized patients with the mentioned conditions.

A standard WHO questionnaire was used to collect the demographic information needed by the children's hospital in Herat City. This WHO questionnaire has been utilized to include questions about the patient's costs and demographic characteristics. In this study, to collect data, considering the inclusion and exclusion criteria, all malnourished children under 5 who were admitted to Herat Children's Hospital were studied during the treatment period. The sampling method is accessible sampling. Because no study has been conducted on the ratio of out‐of‐pocket payments for malnourished pediatric patients in Afghanistan, considering the percentage equal to 0.5 (to determine the maximum sample size) and the error of 0.05 and the power of 80% and accuracy of 0.08, about 300 patients have been selected based on convenience sampling method.

For these patients, an informed consent form was completed. The ethics review board approved the study, adhering to the principles of the Declaration of Helsinki (GR 00699). Informed consent was obtained before enrollment, with written consent from literate participants and thumbprints from illiterate participants. Upon receiving proper authorization and permission from the concerned individual, the caregiver responsible for the child took the initiative to fill out a comprehensive cost questionnaire. The questionnaire aimed to gather detailed information on the expenses incurred while caring for the child. In these questionnaires, information such as demographic characteristics and information related to payment types, especially during the treatment period of malnutrition disease, have been recorded. All patients with Z‐scores less than minus 3 with the aforementioned conditions were included in the study. Demographic variables were obtained by direct questioning and coordinated with patients' records.

For data analysis, the mean and standard deviation were used to describe quantitative variables, while frequency and relative frequency were reported for qualitative variables. All analyses were done using Microsoft Excel 2021 and IBM SPSS statistics V22 software based on the Mann–Whitney test.

## 3 RESULT

This study involved 300 patients under 5 years with severe malnutrition based on inclusion criteria. It was found that 71.3% of the heads of families were illiterate. Based on where the patients live, 90.7% of patients have lived in the rural region (*n* = 272). All the characteristics and descriptive statistics of the studied participants are shown in Table 1.

**Table 1:** Frequency of some demographic characteristics (based on sex) of patients participating in the study.

Demographic characteristics		Frequency	Percent
Place of residence of the patient in terms of urban/rural	Urban	21	7.2
	Rural	272	92.8
Having a house	Yes	192	64
	No	108	36
Education of the head of the family	Illiterate	214	71.3
	Literate	86	28.7
Number of family members	Less than seven	162	54
	More than seven	138	46
The job of the head of the family	Worker	121	41.2
	Employee	26	8.8
	Free job	147	50

The results showed that in families with insufficient income, these patients pay an average amount of 535.48 USD, equivalent to 69% of the treatment costs, directly out of pocket. It should be mentioned that the total amount paid was 776.82 USD. While the families whose income level is sufficient based on their comments, these patients spend an average amount of 535.04 USD, equivalent to 64% of treatment costs, to receive medical services directly out of pocket. It should be noted that the total amount paid was 829.73 USD (Table 2).

**Table 2:** Frequency distribution and percentage and standard deviation of direct out‐of‐pocket payments based on differently affected determinants.

Variable		frequency	Total payment (USD), standard deviation ± mean	Paying OOPE (USD), standard deviation ± mean	Percentage of direct payment out of pocket	The result of the Mann–Whitney test
Different income levels	Not enough	229	776.82 ± 190.52	535.48 ± 137.83	0.69	*Z* = 1.134
	Sufficient	59	829.73 ± 305.36	535.04 ± 143.55	0.64	*p* = 0.257
Number of family members	Less than seven	162	776.05 ± 187.66	526.35 ± 129.14	0.68	*Z* = 1.562
	More than seven	138	799.59 ± 247.61	544.72 ± 148.06	0.68	*p* = 0.118
Education level	illiterate	214	752.29 ± 181.94	534.49 ± 148.94	0.71	*Z* = 4.063
	Literate	86	881.1 ± 275.99	537.79 ± 107.91	0.61	*p* < 0.001
Urban/rural	Urban	21	1014.53 ± 420.86	545.93 ± 122.65	0.54	*Z* = 2.691
	Rural	272	770.22 ± 185.68	534.49 ± 140.14	0.69	*p* = 0.007
Having a house	yes	192	805.42 ± 233.53	538.34 ± 142.89	0.67	*Z* = 0.717
	no	108	769.67 ± 189.97	524.26 ± 126.5	0.68	*p* = 0.473

Abbreviation: OOPE, out‐of‐pocket expenditure.

According to the results, 526.35 USD, 68% of the treatment costs, are paid OOPE directly in households with seven or fewer seven people. It should be mentioned that the total amount paid was 776.05 USD. Meanwhile, families with more than seven people in their household pay an average amount of 544.72 USD, equivalent to 68% of treatment costs, to receive medical services directly out of pocket. It should be mentioned that the total amount paid was 799.59 USD (Table 2).

The results demonstrated that among the illiterate people who were referred to the studied hospital and received services, these people paid an average amount of 534.49 USD, which is equivalent to 71% of the treatment costs, as direct payment out of pocket, it should be mentioned that the total amount paid was 752.29 USD. While educated people visiting the hospital, these people spend an average amount of 537.79 USD, equivalent to 61% of the treatment costs, to receive medical services directly out of pocket; it should be noted that the total amount paid was 881.1 USD (Table 2).

The results also showed that among the residents of Herat City who referred to the studied hospital to receive services, these people paid an average amount of 545.93 USD, which is equivalent to 54% of the treatment costs, as direct payment out of pocket, should be noted that the total amount paid was 1014.53 USD. While the people living in the rural areas of Herat City, referring to the studied hospital, deliver an average amount of 534.49 USD, which is equivalent to 69% of the treatment costs to receive medical services directly out of pocket, it should be mentioned that the total amount paid is 770.22 USD (Table 2).

The results showed that in the families of people who were referred to the studied hospital to receive services, the most reported disease in these families is high blood pressure, and these families pay an average amount of 524.15 USD, which is equivalent to 65% of the treatment costs, directly out of pocket. It should be mentioned that the total amount paid was 822.14 USD. The second disease in these households is tuberculosis, in which 583.88 USD, equivalent to 74% of healthcare costs, is paid directly out of pocket. It should be noted that the total amount paid was 798.82 USD. The third disease in these households is diabetes. Patients pay 68% of healthcare costs, equivalent to 577.22 USD, directly out of pocket. The total amount paid for diabetes was 843.37 USD. The least reported disease in these households is hepatitis (Table 3).

**Table 3:** Frequency distribution and percentage and standard deviation of direct payment OOP according to the type of disease.

Type of medical service based on the sickness	Frequency	Total payment (USD) Standard deviation ± mean	Paying OOPE (USD) Standard amount ± mean	Percentage of direct payment OOP	Standard deviation
Diabetes	19	843.37 ± 204.05	577.22 ± 95.26	0.68	0.153
MI	3	1107.7 ± 728.75	566.83 ± 11.44	0.51	0.315
Cancer	7	824.34 ± 178.09	588.17 ± 121.11	0.71	0.859
Asthma in elderly	6	882.2 ± 132.99	551.32 ± 10.78	0.62	0.175
Asthma in children	4	716.87 ± 94.71	468.82 ± 140.8	0.65	0.139
BP	42	822.14 ± 243.43	524.15 ± 164.01	0.64	0.139
Osteoporosis	12	751.52 ± 218.57	504.35 ± 153.56	0.67	0.178
Tuberculosis	22	798.82 ± 229.68	583.88 ± 142.78	0.73	0.101
Hepatitis	1	561	379.5	0.68	–

Abbreviations: BP, blood pressure; MI, myocardial infarction; OOP, out of pocket; OOPE, out‐of‐pocket expenditure.

According to the results of the surveys, based on the breakdown of the items of medical services received, the direct payment for a doctor's visit is 2.08 USD, equivalent to 3.37%. The highest immediate payment OOPE for pharmaceutical items is 33.66 USD or 54%. The direct out‐of‐pocket expense for these patients is for radiology services, where only 8.8 USD, equivalent to 14.26% of the cost, is paid directly out‐of‐pocket by the patients (Table 4).

**Table 4:** Frequency distribution and percentage and standard deviation of direct payment OOP based on the separation of the type of doctor's visit, pharmaceutical items, radiology, and laboratory expenses.

Type of medical service in detail	Frequency	Amount paid directly out of pocket (USD)	Percentage of direct payment out of pocket	Standard deviation
Examination by a doctor	288	2.08 ± 0.6	0.32	0.095
Medicine	288	33.66 ± 13.09	0.46	0.255
Laboratory services	289	17.16 ± 15.4	0.23	0.200
Radiology services	291	8.8 ± 1.1	0.13	0.154

Abbreviation: OOP, out of pocket.

As the last step, the study's results show that, by analyzing the frequency distribution and the percentage of out‐of‐pocket payments in proportion to the family's estimated income, it is determined that about 93% of the families referring to Herat Children's Hospital have catastrophic expenses due to receiving medical services to treat the disease (Table 5).

**Table 5:** Frequency distribution and percentage of out‐of‐pocket payments in proportion to the family's estimated income.

Total out‐of‐pocket expenses	Frequency	Percentage of direct payment out of pocket
Families with less than 40% of their income spent on the treatment of malnourished children	21	7
Families who spent 40% or more of their income on the treatment of malnourished children	270	93

To explore the factors influencing direct out‐of‐pocket payments, the impact of various variables such as patient residence (urban/rural), home ownership, Education of the head of the family, number of household members, household head income, and presence of underlying diseases on out‐of‐pocket expenses were initially examined using univariate linear regression analysis. Among these factors, the Education of the head of the family and patient residence (urban/rural) were found to be statistically significant. Subsequently, a multiple linear regression analysis was conducted to determine the factors influencing direct payments from patients’ pockets. The research results revealed that the variables patient residence (urban/rural) (−0.082) and household head education level (0.057) significantly influenced out‐of‐pocket payments by patients (*p* < 0.05) (Table 6).

**Table 6:** Estimation coefficient for factors influencing out‐of‐pocket costs for patients.

Parameter	*B*	Standard error	*t*	Significant	95% Confidence interval	
					Lower bound	Upper bound
Place of residence of the patient in terms of urban/rural	−0.082	0.033	−2.522	0.012	−0.147	−0.018
Education of the head of the family	.057	0.019	3.020	0.003	0.020	0.094

## 4 DISCUSSION

Afghanistan is located in the Middle East, with a population of approximately 38 million.^20^ According to data from the World Bank, health expenses in Afghanistan account for approximately 16.83% of the country's gross domestic product (GDP).^21^ Nevertheless, people's access to pharmaceutical services and their ability to receive health services need improvement.^22^ The amount of direct payment from pocket for the services received by patients referring to medical centers and hospitals in this country is considered high. Therefore, this study aims to accurately estimate the out‐of‐pocket payments in hospitalized patients with severe malnutrition in a children's hospital in Herat Province in 2023. Based on the findings and knowledge of the researchers, the present study is the first to investigate and estimate the amount of direct out‐of‐pocket payments in Afghanistan. Researchers and policymakers can welcome its results in Afghanistan and the world. Such studies can help to fill the knowledge gap between researchers and policymakers in this regard.

The first objective of this study was to determine the amount of direct out‐of‐pocket payments at different income levels. The results showed that in families with insufficient income, these patients pay 70% of the treatment costs directly out of pocket. In a study, Sabatino et al. concluded that out‐of‐pocket payments were much more common among women whose families had high incomes. Hence, women pay 14.5% of the cost of mammography directly out of pocket in homes with lower payments. In contrast, 29.5% of mammography expenses are paid out of pocket by women living in high‐income households.^23^ So logically, a significant relationship exists between household income and willingness to pay OOPE for medical treatment. The higher the household income, the higher the desire to pay out of pocket. This category also points to another point. Suppose the per capita income of the household is low, but on the other hand, the government health and treatment system and the insurance of the mentioned country cannot cover the health and treatment costs. In that case, Households with a low‐income level will practically not have an acceptable level of health and treatment facilities, and this itself can impose hidden and obvious overall costs on the economic system of that country's health and treatment system in the medium and long term. A study in Georgia showed that the lack of access to medicines is an essential factor in increasing households' exposure tocatastrophic costs. Covering medication in the public sector significantly reduced the amount of direct payment from the households' pockets.^24^

The second goal of this study was to determine the amount of direct out‐of‐pocket payments according to household dimensions. The results showed that 68% of the treatment costs were paid directly out of pocket in households with seven or fewer than seven people. At the same time, households with more than seven people in their family pay 70% of treatment costs to receive medical services straight out of pocket. In a study conducted in Ethiopia to estimate the amount of direct out‐of‐pocket payments in public hospitals, the results showed with certainty that with the increase in the number of family members, the amount of direct out‐of‐pocket costs to receive medical services increases significantly.^25^ Also, the results of the study by Xu et al. showed that the probability of poor households using private services increases due to the increase in the number of children under 5 years of age who are not employed, which itself requires an increase in the number of direct payments from the households' pockets.^26^

The third objective of this study was to determine the number of direct out‐of‐pocket payments for healthcare services in families with different education levels. The results showed that among the illiterate people referred to the studied hospital to receive benefits, these people paid 71% of the treatment costs as direct payment out of pocket. While educated people visit the hospital, these people deliver 63% of the treatment costs to receive medical services directly out of pocket. Sabatino et al. concluded in their study that with the increase in the level of education in households that refer to healthcare centers and hospitals that provide health services, the percentage of direct out‐of‐pocket payments for receiving health services is lower, so their results showed that in people with a bachelor's degree or higher, these people pay 21.1% of healthcare costs directly out of pocket. In comparison, people with less than a diploma education pay 23.2% of healthcare costs directly out of pocket. The results of these two studies are consistent.^22^

In another systematic review study conducted on patients over 65 years old by Corrieri et al., low‐income people are found to pay the highest OOPE relative to their income. Prescription medicines have the largest share. Lower education is associated with higher OOPE for prescription medicines and a higher likelihood of inadequate insurance support.^23^

The fourth objective of this study was to determine the number of direct payments from the patients' pockets in patients who live in different areas of Herat Province (urban and rural). The results showed that among the people living in Herat City who were referred to the studied hospital and received services, these people paid 59% of the treatment costs as direct payment out of pocket, While the people living in the rural areas of Herat City, referring to the studied hospital, these people deliver 70% of the treatment costs to receive medical services directly out of pocket. Amaya‐Lara, in a study,^27^ concluded that the difference in the occurrence of catastrophic healthcare costs among different regions of Colombia could be due to population distribution in cities and villages. In Colombia, health insurance coverage varies by region: more households with subsidized insurance (57.1%) live in rural areas, while urban areas have fewer covered families than rural areas (40.7%). His study showed that the percentage of catastrophic healthcare expenses in uninsured households was higher than in insured homes in both regions: 7.4% versus 6.9% in urban areas and 14.1% versus 15.5% in rural areas. Likewise, the percentage of homes with catastrophic medical expenses is higher in rural areas than in urban areas in all regions, and in rural areas, this ratio is increasing.^28^

The fifth objective of this study was to determine the number of direct out‐of‐pocket payments based on the type of illness of families referred to health centers and hospitals under investigation. The results showed that the most reported disease is high blood pressure in the families of people referred to the studied hospital for services. The second disease in these households was tuberculosis. The last reported disease in these households is hepatitis. Kaonga et al., in a study,^29^ investigated the amount of direct out‐of‐pocket costs for malaria/fever, respiratory infections, headache, diarrhea, and other diseases. Their study concluded that direct out‐of‐pocket payments for the mentioned diseases are 52.94%, 3.89%, 5.14%, 10.06%, and 27.95% for other diseases. Their examined diseases were different from the diseases reported in this study. Still, the difference that can be seen in these two types of studies is that in the current study, most non‐communicable diseases were reported by those referred to the study hospital. Still, in the study, Kaonga reviewed infectious diseases.^29^

The sixth goal determined in this study was to determine the amount of direct out‐of‐pocket payments based on the type of doctor's visit, pharmaceutical items, radiology, and laboratory expenses. The results showed that based on the breakdown of received medical services, the direct out‐of‐pocket payment for doctor's visits and pharmaceutical items were 32% and 46%, respectively. The lowest out‐of‐pocket cost for these patients was for radiology services (13%). Mercier et al., in a study,^30^ calculated out‐of‐pocket expenses for medical services. The results of his research showed that the amount of direct payment OOPE for the medical visit when the patient goes to the treatment center as an outpatient is 9.9%, the amount of income for diagnostic and laboratory tests is 5.7%, pharmaceutical services is 5.5%, the medical equipment needed for Treatment of the disease is 48.8%, radiotherapy, and chemotherapy services are 22.7%, and the amount of direct out‐of‐pocket payment for other services required for the treatment of the disease is 27.5%.^30^

In other words, in most cases, the pharmaceutical services of patients with most diseases include a more significant proportion of the family's income than the outpatient services they receive. It causes an increase in the amount of direct payment from the patient's pockets. So, it can be said that the governments or the policymakers of the healthcare system of this country should focus a large part of their focus on valid policies in the health and treatment field to reduce the amount of direct payment from patients' pockets to cover the costs related to Medicines and complementary treatments for diseases. The lack of advance payments for health or health insurance is the main reason for the high amount of direct out‐of‐pocket expenses for health care, especially in some developing countries like Afghanistan and Africa, which results in a high percentage of households facing catastrophic health costs. A study in Nigeria conducted to analyze the out‐of‐pocket expenses of families facing devastating health payments shows that 24% of Nigerian households incur catastrophic health costs and charges. The burden is highest among the wealthiest quintile of income in Nigeria. Also, the findings show that direct out‐of‐pocket payments have succeeded in changing the poverty status of households (lowering the home below the poverty line) in families that were initially on or above the poverty line.^31^

A notable finding is the observed disparity in out‐of‐pocket payments between urban and rural areas. Urban residence was associated with lower out‐of‐pocket expenses than rural areas, as indicated by the negative coefficient (−0.082). This difference may reflect disparities in healthcare infrastructure, service availability, and socioeconomic factors between urban and rural settings. Limited access to healthcare facilities and services in rural areas could result in higher out‐of‐pocket expenses for patients seeking care, exacerbating the financial burden on households already facing economic challenges. Two studies in Benin and Peru, low‐income countries, showed the same results, proving that it is essential to focus on increasing the quantity and quality of health services in rural areas.^32^, ^33^ The problem is that in rural areas, a vast majority of residents suffer from poverty, and catastrophic health expenditures may result in tremendous health issues. In addition, the positive relationship between household head education level and out‐of‐pocket payments highlights the influence of education on healthcare utilization and expenditure. Higher education levels among household heads were associated with increased out‐of‐pocket expenses, as evidenced by the positive coefficient (0.057). Mehrara and colleagues previously demonstrated the effect of household education level on health expenditures.^34^, ^35^, ^36^, ^37^ This finding suggests that households with better‐educated heads may have more significant health awareness and access to healthcare services, leading to higher health expenditure. It underscores the importance of health literacy and education in shaping healthcare‐seeking behaviors and financial decisions related to healthcare expenses.^38^

This research study stands out for its significant contribution to the field, as it sheds light on the delicate matter of direct payment from the pockets of residents in one of the largest cities in Afghanistan. The study is unique in its approach and provides valuable insights that were previously unavailable. The results of this study can lead to planning and necessary policies regarding the required activities related to the health system in Afghanistan; however, this study, like many survey studies, also has areas for improvement. One of its weak points is that the data on direct payment from the pockets of the population of Herat City was collected only at a specific time. If the study was conducted during four seasons of the year, the results might change a little. Another weakness of the study is that this study was conducted on hospitalized patients suffering from severe malnutrition in a children's hospital in the Herat Province. The generalizability of the study to direct payment from the pockets of all patients may need to be revised. If we had increased the sample size in the general departments of Herat City hospitals, the results might have been different.

## 5 CONCLUSIONS

This study shows that OOPE in Afghanistan, a country with a weak and underdeveloped economy, where a high malnutrition rate in children under 5 years of age is observed. A high rate of complications and high infant mortality have been identified in it, which is increasing. High OOPE is a significant barrier to quality healthcare services and access to appropriate and affordable medicines. More research is needed to examine the pricing mechanisms of essential medicines, consumer preferences for purchasing, and provider preferences for prescribing. To implement justice by benefiting from adequate health and treatment services and supporting the weak and vulnerable underprivileged sections of Afghan society, geographical imbalances and broad health financing options such as government payments, incentives, and health insurance should be considered. Considering that the new sector plan implemented this year has a central component, providing a package of essential services to improve universal health coverage, our findings can help refine and validate the national action plan and the national child health strategy.

## AUTHOR CONTRIBUTIONS

**Amirmohammad Tajik**: Investigation; writing—original draft; writing—review and editing; methodology; formal analysis; software. **Vahid Ghavami**: Methodology; validation; software; formal analysis; data curation. **Shakib Popal**: Investigation; conceptualization. **Hamidreza Shabanikiya**: Methodology; validation; software; formal analysis; data curation. **Mehdi Varmaghani**: Methodology; conceptualization; validation; visualization; writing—review and editing; project administration; formal analysis; supervision; resources.

## CONFLICT OF INTEREST STATEMENT

The authors declare no conflict of interest.

## ETHICS STATEMENT

The study has been approved by the Ethics Committee of Mashhad University of Medical Sciences with the code of IR.MUMS.REC.1399.322. All participants provided written informed consent forms for the various aspects of data collection. It was also implemented following the principles and regulations of confidentiality and privacy. All research methods were carried out following relevant guidelines and rules.

## TRANSPARENCY STATEMENT

The lead author Mehdi Varmaghani affirms that this manuscript is an honest, accurate, and transparent account of the study being reported; that no important aspects of the study have been omitted; and that any discrepancies from the study as planned (and, if relevant, registered) have been explained.

## Data Availability

The data that support the findings of this study are available on request from the corresponding author. The data are not publicly available due to privacy or ethical restrictions. Dr. Mehdi Varmaghani, the corresponding author, had full access to all of the data in this study and takes complete responsibility for the integrity of the data and the accuracy of the data analysis
